# pH‐mediated activation of the lysosomal arginine sensor SLC38A9

**DOI:** 10.1002/1873-3468.70352

**Published:** 2026-05-03

**Authors:** Xuelang Mu, Ampon Sae Her, Tamir Gonen

**Affiliations:** ^1^ Department of Biological Chemistry University of California Los Angeles CA USA; ^2^ Department of Physiology University of California Los Angeles CA USA; ^3^ Molecular Biology Institute University of California Los Angeles CA USA; ^4^ Howard Hughes Medical Institute University of California Los Angeles CA USA

**Keywords:** amino acid transport, mTOR complex, pH‐regulation, SLC family, transceptor

## Abstract

Cells rely on metabolic control; the mechanistic target of rapamycin complex 1 (mTORC1) senses nutrient availability, particularly amino acids. Lysosomes maintain amino acid homeostasis through recycling. SLC38A9, a lysosomal amino acid transporter, functions as a critical sensor in the mTORC1 pathway. Here, we investigate how pH regulates SLC38A9 activity. We show that arginine uptake is pH‐dependent, with His544 residue serving as the pH sensor. Mutating His544 abolishes pH dependence without impairing overall transport, indicating His544 influences transport through protonation/deprotonation, instead of involving in the substrate binding. We propose a working model for pH‐induced activation, through comparing two determined SLC38A9 structures at different pH. These findings reveal how local ionic shifts regulate lysosomal transporters and fine‐tune SLC38A9 function to control mTORC1 signaling.

## Graphical abstract

Cells monitor nutrient levels via the lysosomal transporter SLC38A9 to activate the mechanistic target of rapamycin complex 1 (mTORC1). This study reveals that SLC38A9 function is regulated by pH. We identified histidine 544 as a critical pH sensor that undergoes conformational changes to control amino acid efflux from lysosomes; therefore, it coordinates metabolic signaling with the cellular environment.

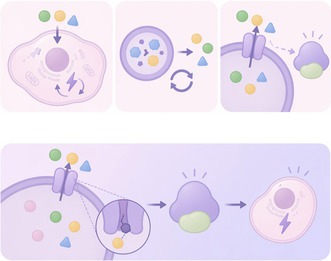

## Abbreviations

**AdiC**, arginine‐agmatine antiporter

**cryo‐EM**, Cryo‐electron microscopy

**drSLC38A9**, *Danio rerio* (zebrafish) SLC38A9

**mTORC1**, Mechanistic target of rapamycin complex 1

**PDB**, Protein Data Bank

***pK*_a_**, acid dissociation constant

**Rag GTPase**, Ras‐related GTP‐binding proteins

**SLC38A9**, solute Carrier Family 38 Member 9

**TM**, transmembrane helix

**V‐ATPase**, vacuolar ATPase

Sensing the environment is essential for cells to coordinate their metabolic activities. Cells must respond to diverse cues, including nutrients, energy levels, and growth factors. A central regulator of this process is the mechanistic target of rapamycin complex 1 (mTORC1), a key hub in cellular metabolism. mTORC1 integrates fluctuations in extracellular and intracellular nutrients and directs cellular outcomes, promoting anabolic processes such as protein, lipid, and nucleotide synthesis, or initiating catabolic processes such as lysosome biogenesis and autophagy [1]. Among these signals, amino acids serve as fundamental cues for the mTORC1 pathway, and maintaining a balance between amino acid synthesis and degradation is critical [2]. Lysosomes function as recycling centers, generating nutrients and storing amino acids. They sustain a low pH to enable the breakdown of proteins, polysaccharides, lipids, and nucleic acids. Consequently, there is a close relationship between lysosomal pH and its functions. Importantly, many transporters are embedded in the lysosomal membrane and operate within the pH gradient between the lysosome and the cytosol [3, 4, 5].

SLC38A9 is a low‐affinity lysosomal amino acid transporter that functions as an upstream amino acid sensor for mTORC1 activation [6, 7, 8]. In addition to sensing, it mediates the efflux of several essential amino acids, including leucine and glutamine, in an arginine‐dependent manner [9]. Thus, SLC38A9 acts as a transceptor, combining the roles of both transporter and receptor [10, 11]. Belonging to the amino acid/auxin permease family, it is characterized by 10–11 transmembrane domains [12] and is also classified within the sodium‐coupled amino acid transporter family [6, 7]. The zebrafish ortholog (Danio rerio; drSLC38A9) shares 61.9% sequence identity and 86.6% similarity with the human protein. Crystal structures of drSLC38A9 revealed 11 transmembrane helices, with the N terminus facing the cytosol and the C terminus positioned in the lysosomal lumen [13]. The arginine‐bound structure identified a substrate‐binding pocket buried among the transmembrane helices [13]. In contrast, the arginine‐free structure showed the N terminus forming a β‐hairpin that lodged within the transporter and occupied the arginine‐binding site [14]. These findings led to a proposed “ball‐and‐chain” model to explain amino acid sensing and signaling by SLC38A9. Complementary cryo‐EM studies of an N‐terminal peptide of SLC38A9 bound to Rag GTPases revealed how the N terminus regulates nucleotide state switching in the GTPases [15]. Together, these results suggest that under nutrient‐rich conditions, when luminal arginine levels are high, arginine displaces the N‐terminal domain from the binding pocket, freeing it to interact with Rag GTPases and activate mTORC1 [14].

Several lysosomal membrane proteins are known to be regulated by pH [16, 17, 18, 19, 20]. This regulation is particularly common at the lysosomal membrane due to the steep pH gradient across it: approximately pH 7.2 in the cytoplasm versus pH 4.7 inside the lysosomal lumen [21]. Because SLC38A9 functions at the lysosomal membrane, we hypothesized that it too may be subject to pH regulation. Proteins regulated by pH, such as transporters and pH‐sensitive enzymes, often contain amino acid residues with side chains that can donate or accept protons depending on the local microenvironment [22, 23]. Among these, histidine (pKa ~ 6.2 in solution) is particularly well known for its role in pH sensing [24, 25]. The imidazole side chain of histidine can either gain or lose a proton depending on the surrounding pH, enabling histidine residues to act as molecular pH sensors in a wide range of biochemical processes. Indeed, many pH‐sensitive systems rely on histidine residues as key regulators [26, 27, 28, 29, 30, 31].

In this study, we examined the pH dependence of SLC38A9 transport activity using purified protein reconstituted into vesicles and radiolabeled ligand uptake assays. By measuring [^3^H]‐arginine uptake across different pH conditions, we found that SLC38A9‐mediated transport is strongly pH‐dependent. Sequence analysis combined with site‐directed mutagenesis identified His544 as a key pH‐sensing residue in human SLC38A9. Based on these results and supporting structural analysis, we propose a working mechanism in which His544 acts as a pH‐regulated allosteric site, modulating arginine transport in response to intracellular pH fluctuations.

## Materials and methods

### Generation of SLC38A9 variants

The sequence encoding hSLC38A9 (Genbank Q8NBW4) was cloned into the *p*Fastbac 1 vector (Invitrogen, Carlsbad, CA, USA) with a sequence encoding a C‐terminal 8X His tag and a thrombin cleavage site. Site‐directed mutagenesis was carried out using a standard protocol involving two PCR steps. All SLC38A9 histidine mutants were confirmed by whole‐plasmid sequencing. Verified plasmids were transformed into DH10Bac cells (From Thermo Fisher Scientific, Carlsbad, CA, USA; MAX Efficiency DH10Bac competent cells) for preparation of bacmids. Recombinant baculovirus was generated and used for transfection following the protocol for the Bac‐to‐Bac Baculovirus Expression System (Invitrogen). Wild‐type SLC38A9 and histidine mutants were overexpressed in *Spodoptera frugiperda* sf‐9 insect cells, which were harvested at 60 h after P2 virus infection. The *Spodoptera frugiperda* sf‐9 insect cells are from expression systems, adapted in ESF 921.

### Protein preparation

Harvested cell pellets were resuspended in lysis buffer containing 20 mm Tris (pH 8.0) and 150 mm NaCl and supplemented with cOmplete Protease inhibitor Cocktail (Roche, Indianapolis, IN, USA), and 10 mm EDTA, 10 mm EGTA, and 10 mm DTT. Eighty homogenizing cycles were then carried out to break cells on ice, followed by ultracentrifugation at 130 000× ***g*** for 40 min. The pelleted membrane was then resuspended and washed with the high salt buffer containing 20 mm Tris pH 8.0 and 1.6 m NaCl followed by ultracentrifugation at 130 000× ***g*** for 30 min. The wash step was repeated twice. The pellets were then resuspended in the lysis buffer, frozen in liquid nitrogen, and stored at −80°C until further use.

To purify SLC38A9 protein and its variants, the membrane fraction was thawed and solubilized with 2% n‐dodecyl‐β‐*d*‐maltopyranoside (DDM; Anatrace, Maumee, OH, USA), 0.2% Cholesteryl Hemisuccinate Tris salt (CHS; Anatrace) in 20 mm Tris pH 8.0, and 150 mm NaCl with 10% glycerol for 2 h at 4 °C. Following another ultra‐centrifugation at 130 000× ***g*** for 45 min, the supernatant was loaded onto TALON Metal Affinity Resin (Clontech, San Jose, CA, USA) and incubated at 4 °C overnight. The resin was washed by 25X column volumes of 20 mm imidazole in a high‐salt buffer containing 20 mm Tris pH 8.0, 500 mm NaCl, 0.2% DDM. Then, the protein was eluted with an elution buffer containing 300 mm imidazole, 0.4% decyl‐β‐*d*‐maltoside (DM), 0.02% DDM, 20 mm Tris pH 8.0, and 150 mm NaCl. The eluted proteins were applied to a Superdex‐200 column (GE Healthcare, Chicago, IL, USA) in a buffer containing 20 mm Tris 8.0, 150 mm NaCl, and 0.2% DM. The peak fraction was collected and concentrated for proteoliposome reconstitution and transport assays.

### Preparation of proteoliposomes

Chloroform‐dissolved chicken egg phosphatidylcholine (egg‐PC; Avanti Polar Lipids, Alabaster, AL, USA) was evaporated using dry nitrogen to yield a lipid film in a small glass vial and further dried under vacuum overnight. The lipids were hydrated in the inside buffer (20 mm Tris 8.0, 90 mm KCl, 10 mm NaCl) at 25 mg·mL^−1^ by vortex for 3 min and then aged at room temperature for 1 h. Liposomes were clarified by five rounds of freezing and thawing in liquid nitrogen and extruded through a 100‐nm membrane with 21 passes (MilliporeSigma, Burlington, MA, USA). The liposomes were pre‐incubated with 1% n‐octyl‐b‐*d*‐glucoside (β‐OG) and 1 mm DDT for 1 h at 4 °C before protein reconstitution. Purified wild‐type SLC38A9 and variants were incorporated at a 1 : 100 (w/w) ratio into destabilized liposomes for 1 h in the 4 °C rotator. Glycerol‐supplemented protein buffer was used in lieu of SLC38A9 protein in liposome‐only control groups. The detergents were removed by incubation overnight with 200 mg per reaction Bio‐Beads, and the proteoliposomes were further incubated with 40 mg per reaction fresh Bio‐Beads for an additional hour. The proteoliposomes and liposome‐only controls were collected using ultracentrifuge at 100 000× ***g*** for 30 min at 4 °C and then resuspended in an outside buffer (100 mm NaCl, different pH buffer: 20 mm Mes 5.5, Mes 6.0, Mes 6.5, Tris 7.0, Tris 7.5 and Tris 8.2) to final lipid concentration of 40 μg·μL^−1^.

### Transport assay

Transport reactions were initiated by adding 0.5 μm L‐[^3^H]‐arginine (batch/Lot #220412; American Radiolabeled Chemicals, Inc, Saint Louis, MO, USA) to 50 μL of proteoliposomes. Assays of liposome‐only controls were carried out in parallel to experimental groups as negative controls. All buffers were chilled, and assays were performed at a 30 °C metal bath. For time‐course uptake assay, at various time points, proteoliposomes were filtered, washed by 10 mL of ice‐cold wash buffer (outside buffer with 10 mm unlabeled L‐arginine), and collected on 0.22 μm nitrocellulose membranes (Millipore) which had been pre‐wet by washing buffer. After washing, each membrane was dried by vacuum for exactly 1 min and transferred into a glass vial with 10 mL scintillation fluid for counting. A time course profile indicates that the retained radio‐ligands reached saturation after 10 min. For the pH‐dependent uptake assay, the equilibrium transport of arginine was measured at 10 min after adding 0.5 μm L‐[^3^H]‐arginine with proteoliposomes. The reaction was quickly placed into 0.22 μm nitrocellulose membranes (Millipore) and washed with the 10 mL ice‐cold wash buffer (which had the same pH as the outside buffer with 10 mm unlabeled L‐arginine). The membranes were collected into a glass vial for liquid scintillation counting. Non‐specific adsorptions of L‐[^3^H]‐arginine by liposomes‐only controls were subtracted from experimental measurements. All experimental and control groups were repeated two to three times.

## Results

### Purified SLC38A9 transports [ ^3^H]‐arginine *in vitro* and is pH regulated

**Fig. 1:**
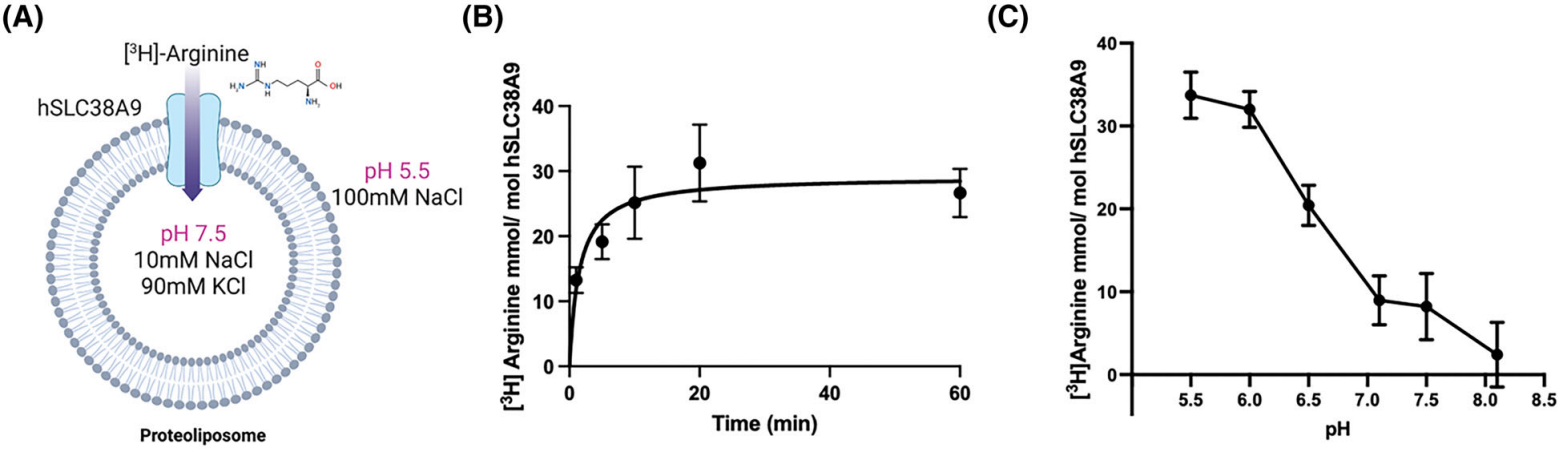
[^3^H]‐arginine uptake assays of SLC38A9 proteoliposomes. (A) Model representation of SLC38A9 proteoliposomes used in this study. (B) Uptake assay in SLC38A9‐reconstituted liposomes showing the efficiency of arginine transport with pH_in_ = 7.5 and pH_out_ = 5.5. This mimics the lysosome‐cytosol pH gradients in the cell. Transport was calculated by subtracting the radioactivity associated with empty liposome controls. Error bars, s.e.m. from three independent proteoliposome preparations; *n* = 3 biological replicates. (C) Effect of pH on the reconstituted SLC38A9. Reconstitution and transport assay were performed at the indicated pH_out_. Transport was calculated by subtracting the radioactivity associated with empty liposomes. Error bars, s.e.m. from three independent proteoliposome preparations; *n* = 3 biological replicates.

SLC38A9 was expressed and purified as described in the methods section. Purified SLC38A9 was reconstituted into proteoliposomes at a neutral internal buffer (pH_in_ 7.5) and contained an internal sodium concentration of 10 mm and potassium concentration of 90 mm. This buffer condition mimics the cytosolic environment [21]. Proteoliposomes were then exchanged into an external buffer with acidic pH (pH_out_ 5.5) and a sodium concentration of 100 mm, containing [^3^H]‐arginine as substrates (Fig. 1A). Under physiological conditions, the lysosomal lumen maintains a high sodium concentration, ranging from 20 to 140 mm [32], similar to the extracellular environment, whereas the cytosol maintains a low sodium concentration of approximately 10–15 mm [33]. The chosen concentrations of sodium (10 mm internal/100 mm external) in the experiment conditions are consistent with established physiological values for the cytosol and lysosomal lumen, respectively. Specifically, the high external sodium mimics the 100–140 mm Na + concentration found within the lysosomal lumen. This sets up a pH and salt to facilitate arginine transport via SLC38A9 until saturation was observed at *t* = 10 min (Fig. 1B). To investigate the effect of pH on the transport activity of SLC38A9, we measured the uptake of [^3^H]‐arginine under different external buffer pH (pH_out_) at pH 5.5, 6.0, 6.5, 7.0, 7.5, and 8.2, while maintaining the inside buffer pH (pH_in_) consistent at pH 7.5. These experiments demonstrated that the uptake of [^3^H]‐arginine was highest at the external pH at 5.5 and 6.0 suggesting that SLC38A9 is indeed pH regulated (Fig. 1C).

### Histidine 544 is identified as the pH sensor

The identification of a pH sensor is critical for elucidating the molecular mechanism underlying the pH‐dependent transport activity of SLC38A9. A *bona fide* pH sensor must undergo pH‐induced changes, either to mediate new interactions or to abolish existing interactions, thereby modulating substrate transport. Such pH‐induced changes are typically mediated by the protonation and deprotonation of ionizable amino acid residues, such as histidine, glutamic acid, and aspartic acid [23]. Given that SLC38A9 is activated at pH values of 5.5–6.0, the amino acid histidine, with a pK_a_ of ~ 6.2, emerges as a plausible pH sensor, as it can undergo reversible protonation under these conditions.

**Fig. 2:**
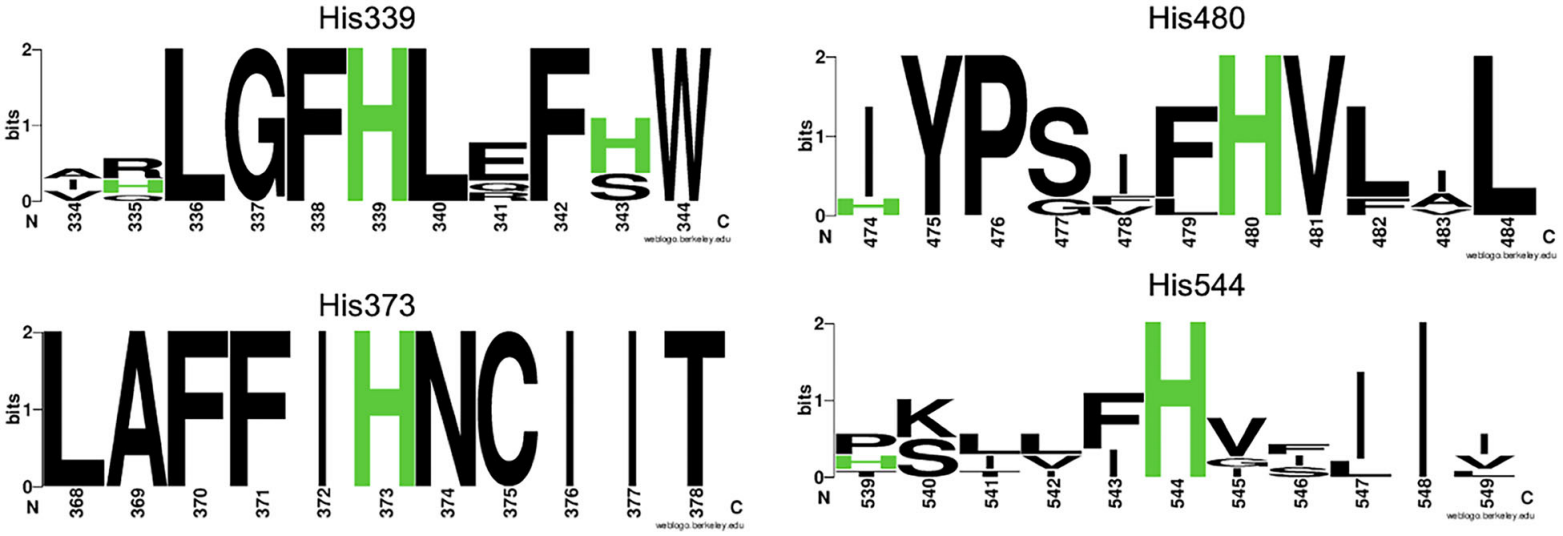
The consensus logo plot for conserved histidine residues. The logo plot was generated from the multiple sequence alignment of six species of SLC38A9 from the UniProt database. His339, His373, His480, and His544 are shown to be highly conserved among different species of SLC38A9.

We proposed that a functionally important histidine residue may be evolutionarily conserved. We conducted a sequence alignment of SLC38A9 protein sequences from six different species (*Danio rerio* (Zebrafish), *Rattus norvegicus* (Rat), *Mus musculus* (Mouse), *Homo sapiens* (Human), *Xenopus laevis* (African clawed frog), and *Xenopus tropicalis* (Western clawed frog)) to identify conserved histidine residues (Fig. 2). Four histidine residues (His339, His373, His480, and His544) within the human SLC38A9 protein are conserved across five different species, as shown in green in the protein sequence alignment (Fig. 2). Consequently, these four histidine residues are prime candidates for pH‐regulatory functions.

**Fig. 3:**
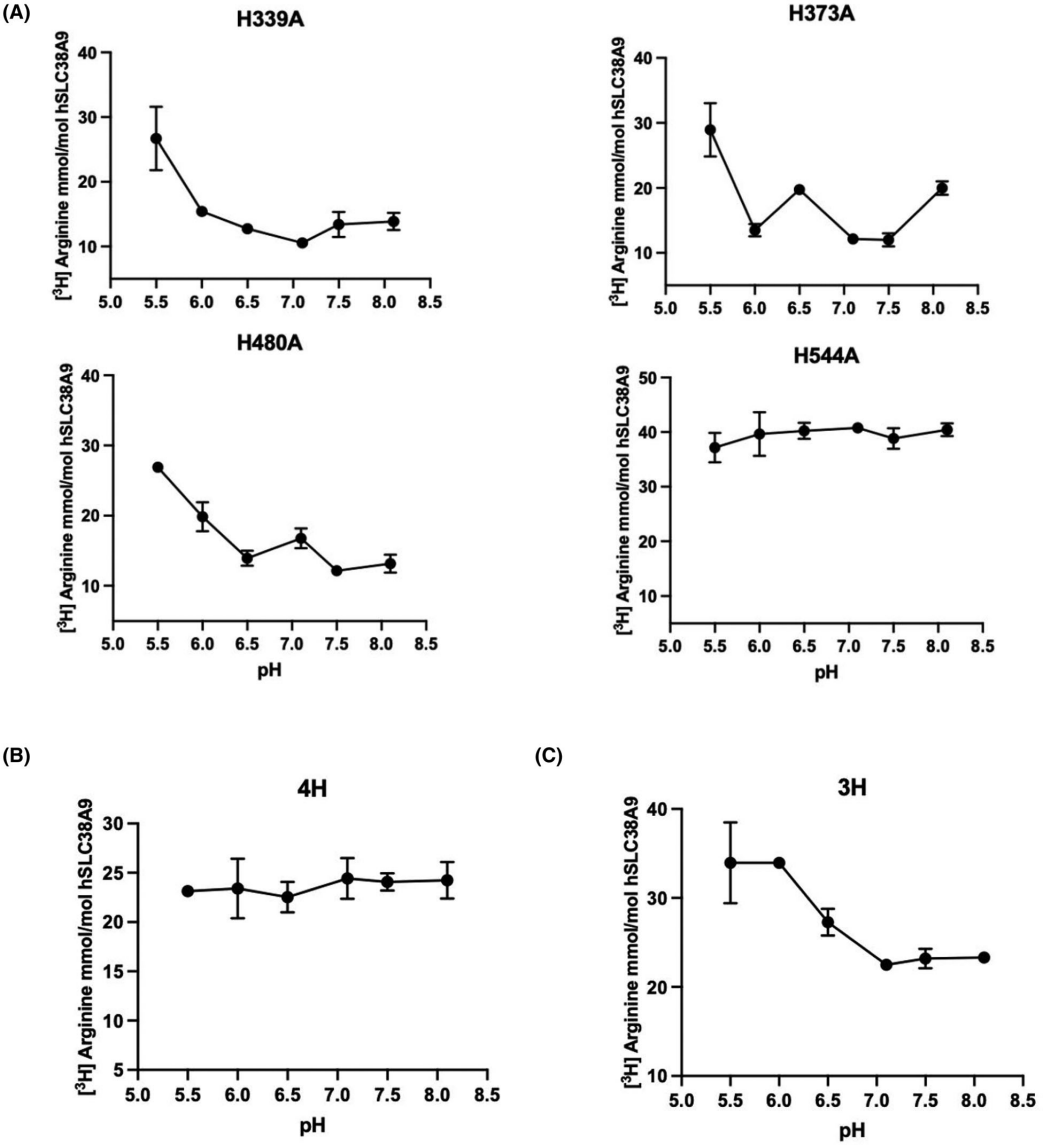
[^3^H]‐arginine uptake assays of histidine mutants of SLC38A9. (A) Effect of pH on single site mutants of SLC38A9 (H339A, H373A, H480A, and H544A) in reconstituted proteoliposomes. All mutants (H339A, H373S, H480A) but H544A show pH dependency where the maximum activity is at pH 5.5 and decreases with higher pH. Error bars, s.e.m. from three independent proteoliposome preparations; *n* = 3 biological replicates. Empty liposome controls were subtracted from these final arginine uptake graphs. (B) Effect of pH on the quadruple site mutants of SLC38A9 (H339A, H373A, H480A, and H544A) in reconstituted proteoliposomes. Error bars, s.e.m. from three independent proteoliposome preparations; *n* = 3 biological replicates. Empty liposome controls were subtracted from these final arginine uptake graphs. (C) Effect of pH on the triple site mutants of SLC38A9 (H339A, H373A, and H480A) in reconstituted proteoliposomes. Error bars, s.e.m. from three independent proteoliposome preparations; *n* = 3 biological replicates. Empty liposome controls were subtracted from these final arginine uptake graphs.

Next, we generated mutants of SLC38A9, in which each of these histidine residues (His339, His373, His480, and His544) were individually mutated to alanine, resulting in mutants designated as H339A, H373A, H480A, and H544A. Importantly, each of these mutants demonstrated functional proficiency, as evidenced by their ability to facilitate the transport of [^3^H]‐arginine into proteoliposomes. Importantly, all mutants also displayed pH sensitivity with the exception of H544A. The mutants H339A, H373A, and H480A displayed pH‐dependent regulation of [^3^H]‐arginine uptake where the maximum activity occurred at the external pH of 5.5. In contrast, when Histidine 544 was mutated to alanine (H544A), the [^3^H]‐arginine uptake remained unaffected by external pH variations, indicating the loss of pH‐regulatory capabilities in the H544A SLC38A9 mutant (Fig. 3A).

To further verify whether H544 is the pH sensor, we engineered two additional mutants: a quadruple‐site mutant containing all four histidine mutations (i.e. H339A, H373A, H480A, and H544A; referred to as the 4H mutant) and a triple‐site mutant comprising H339A, H373A, and H480A mutations (referred to as the 3H mutant). While the 4H mutant showed abolished pH sensitivity, the 3H mutant maintained pH sensitivity (Fig. 3B,C, respectively). These results further suggest that H544 acts as the pH sensor in SLC38A9.

### SLC38A9 is regulated by pH and coupled to Na^+^ transport

**Fig. 4:**
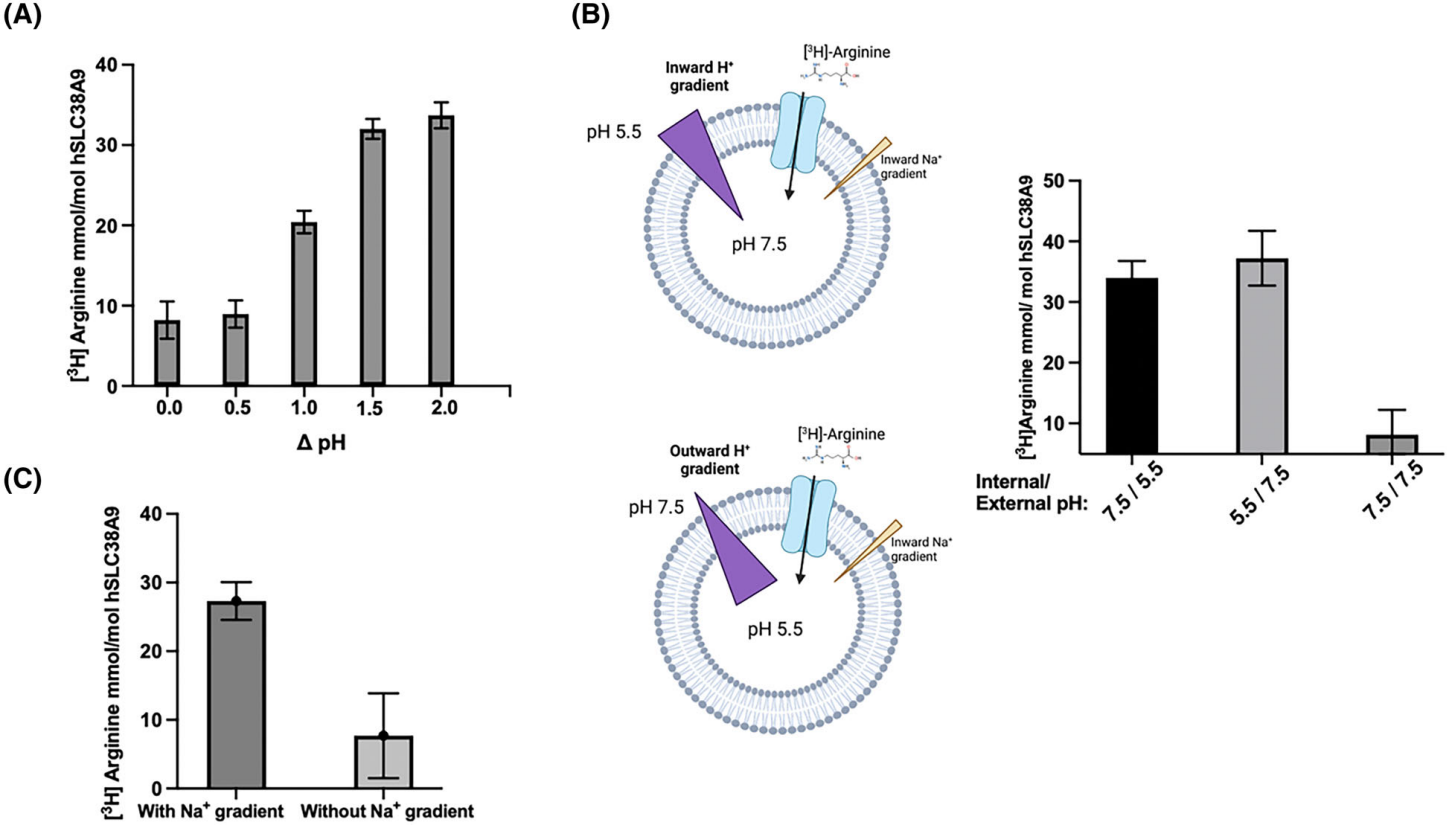
[^3^H]‐arginine uptake assays of SLC38A9 proteoliposomes in different pH gradients and Na^+^ gradients. (A) The equilibrium uptake of [^3^H]‐arginine by SLC38A9 proteoliposomes varied with the differences between pH_in_ and pH_out_. Error bars, s.e.m. from three independent proteoliposome preparations; *n* = 3 biological replicates. Empty liposome controls were subtracted from these final arginine uptake graphs. (B) [^3^H]‐arginine uptake activity of SLC38A9 proteoliposomes in the presence of an inward versus outward H^+^ gradient versus symmetric pH. All the experiments had an inward Na^+^ gradient. Error bars, s.e.m. from three independent proteoliposome preparations; *n* = 3 biological replicates. Empty liposome controls were subtracted from these final arginine uptake graphs. (C) [^3^H]‐arginine uptake assays of SLC38A9 with and without Na^+^ gradient. Error bars, s.e.m. from three independent proteoliposome preparations; *n* = 3 biological replicates. Empty liposome controls were subtracted from these final arginine uptake graphs.

The transport activity of wild‐type SLC38A9 varied significantly under different pH conditions across liposomal membranes. The uptake of [^3^H]‐arginine increased substantially when a pH gradient was present, whereas uptake was minimal in the absence of a pH difference (Fig. 4A).

While SLC38A9 is known to be a sodium‐coupled lysosomal transporter [6, 34], the exact role of the transmembrane proton gradient in driving or just regulating its transport cycle remains unclear.

To determine whether SLC38A9 is directly coupled to the proton gradient or if its function is merely influenced by pH variations, we examined [^3^H]‐arginine uptake in the presence of reversed proton gradients across liposomal membranes. Under an inward proton gradient (pH 7.5 internal/pH 5.5 external), SLC38A9‐mediated uptake of [^3^H]‐arginine was observed. Reversing the proton gradient to outward (pH 5.5 internal/pH 7.5 external) did not abolish [^3^H]‐arginine uptake. However, when both internal and external pH were symmetrically set to 7.5 (meaning no pH gradient), [^3^H]‐arginine uptake was markedly reduced (Fig. 4B). These findings suggest that arginine uptake by SLC38A9 is not dependent on the direction of the proton gradient, as both inward and outward gradients can enhance [^3^H]‐arginine uptake.

Additionally, these results highlight the essential role of an acidic environment on the one side of the vacuolar membrane in supporting human SLC38A9 activity. In drSLC38A9, we observed a transport pattern similar to that of SLC38A9. The maximum uptake of [^3^H]‐arginine occurred at an internal pH of 7.5 and an external pH of 6.5 (Fig. S1). However, when the external pH was raised to alkaline levels (at an external pH of 7.5 and 8.0), [^3^H]‐arginine uptake remained consistently low, regardless of the presence of a proton gradient. These findings confirm that [^3^H]‐arginine transport by SLC38A9 is not solely driven by a proton gradient but requires an acidic pH on the one side of the membrane for efficient uptake.

SLC38A9 is a sodium‐coupled transporter [6, 7]. Previous studies using liposomes reconstituted with drSLC38A9 in various cationic buffers found that the presence of sodium significantly enhanced arginine uptake compared to the presence of potassium [13]. This observation prompted us to investigate whether SLC38A9 relies on sodium gradients across liposomal membranes for its function. To address this, we established conditions with a low internal sodium concentration (10 mm) and a high external concentration (100 mm). Under these conditions, SLC38A9‐reconstituted proteoliposomes exhibited substantial [^3^H]‐arginine uptake (Fig. 4C). In contrast, when there was no sodium gradient across the liposomes (with 100 mm NaCl inside and outside), [^3^H]‐arginine uptake was negligible in SLC38A9‐containing proteoliposomes, even in the presence of an optimal pH gradient (pH 7.5 inside and pH 5.5 outside) (Fig. 4C). These findings suggest that a sodium gradient is a significant energetic contributor to arginine transport in our *in vitro* model. While the proton gradient clearly serves as a critical allosteric regulator, further studies are needed to determine the relative thermodynamic contributions of sodium versus protons in the complex environment of the native lysosomes.

### pH‐sensing mechanism of His544

Our experimental evidence suggests that His544 may function as a pH sensor in SLC38A9 and that protonation or deprotonation of His544 may influence the ability of the substrate to bind to SLC38A9 and to be transported.

**Fig. 5:**
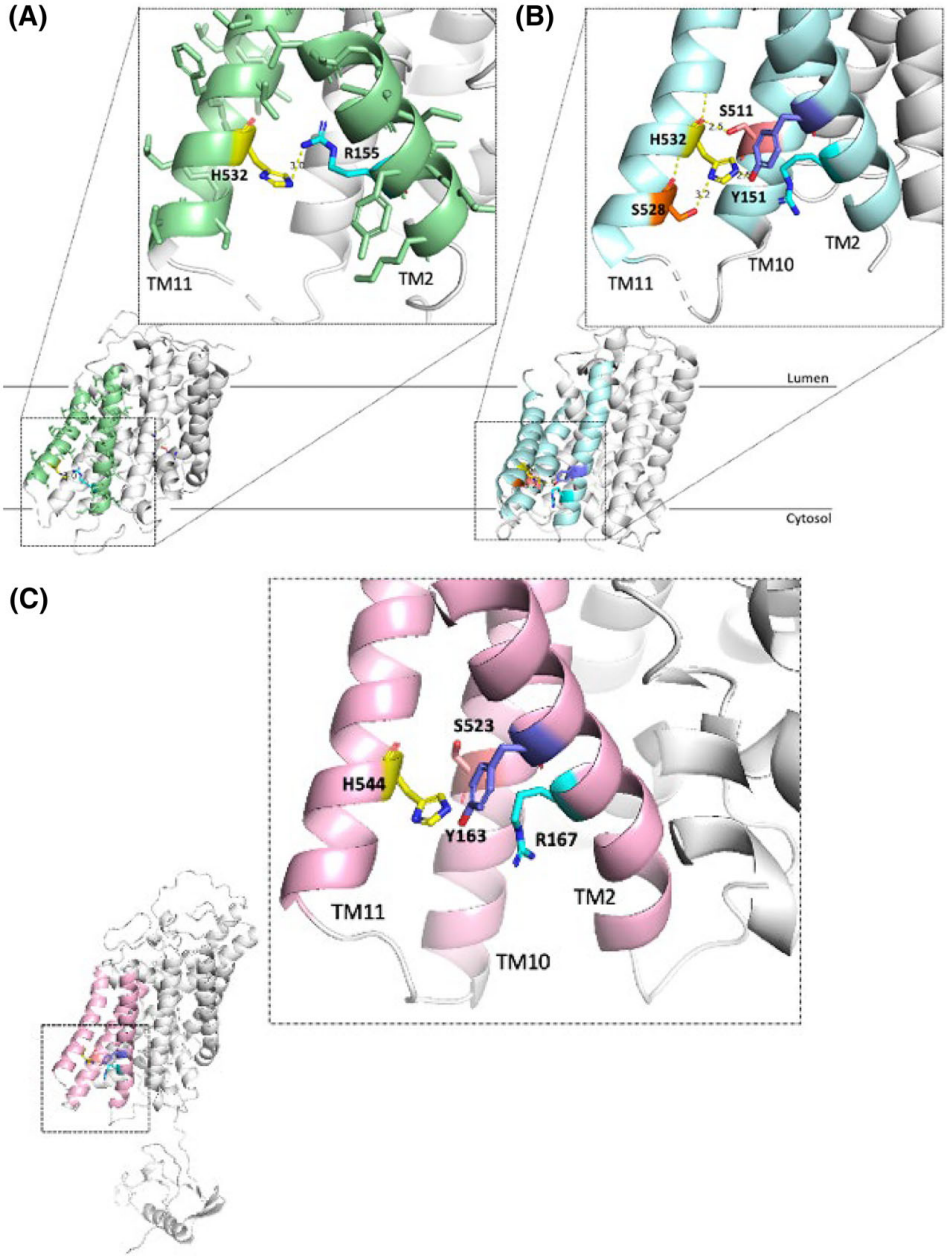
pH‐sensing mechanism of His544 in hSLC38A9. The interactions between His532 and its surrounding residues in drSLC38A9 shift dynamically with the protonation and deprotonation of His532. (A) In the cytosol‐open state structure of drSLC38A9 with bound substrate arginine (PDB: 6C08), deprotonated His532 interacts exclusively with Arg155 from TM2. His532 is shown in yellow, while Arg155 is shown in cyan. (B) In substrate arginine‐free structure of drSLC38A9 (PDB: 7kgv), protonated His532 tightly interacts with various surrounding residues: Tyr151 (pink) from TM2, Ser511 (purple) from TM10, Ser528 (orange) from TM11. The Arg155, as shown in cyan, was too far away from interacting with His532. (C) The alphafold model of hSLC38A9 reveals that His544 is surrounded by conserved residues that may undergo interaction shifts with His544 during its protonation and deprotonation. His544 is shown in yellow, Tyr163 in purple, Arg167 in cyan, and Ser523 in pink.

This hypothesis is supported by structural analysis of drSLC38A9 crystal structure [13, 14], one of which was determined at pH 7.2 and the other at pH 6.0. The substrate‐bound crystal structure of drSLC38A9, solved at pH 7.2 [13], reveals that His532—the homologous residue of human SLC38A9 His544—interacts with Arg155 from TM2 through a cation‐pi interaction between the neutral His and the cation Arg^+^ (Fig. 5A). In contrast, the drSLC38A9 crystal structure which was obtained at pH 6.0 (an acidic condition that facilitates transport) captured the transporter in a substrate‐free conformation [14] without bound arginine. In this state, protonated His532 formed interactions with Tyr151 from TM2 and Ser511 from TM10, indicating a shift in its interaction network compared to the high‐pH structure (Fig. 5B). These distinct pH‐dependent interactions suggest that TMs may undergo conformational rearrangements leading to local structural changes that facilitate gate opening. Notably, the structural flexibility of TM2 and TM10 is consistent with their established role in the arginine transport cycle of a related transporter, AdiC arginine transporter, where their movement modulates substrate occlusion within the transporter [35].

Given the high sequence conservation between hSLC38A9 and drSLC38A9, it is likely that His544 undergoes analogous protonation‐dependent interaction shift, facilitating conformational transitions in hSLC38A9. Structural modeling using alphafold supports this postulate, showing that His544 in hSLC38A9 is surrounded by Arg167 and Tyr163 from TM2 and Ser523 from TM10 (Fig. 5C). This suggests that under high‐pH conditions, His544 may preferentially interact with Arg167, whereas under low‐pH conditions, it may form interactions with Tyr163 and Ser523. Furthermore, the conservation of His544 and its associated interactions across species indicates that this histidine residue functions as a conserved pH sensor in SLC38A9 homologs.

## Discussion

The lysosome is a membrane‐bound organelle responsible for degrading biological macromolecules delivered through endocytic, phagocytic, and autophagic pathways [36, 37]. Its lumen contains more than 60 hydrolases, most of which function optimally in an acidic environment [38, 39]. To support their activity, the lysosomal pH is tightly maintained between 4.5 and 5.0 [40, 41], in contrast to the cytosolic pH of 7.0–7.5 [42, 43]. This steep gradient is established and sustained by the vacuolar ATPase (V‐ATPase), which actively pumps protons into the lysosome [44, 45]. Beyond enabling hydrolase function, the 100‐ to 1000‐fold proton gradient also provides the driving force for many lysosomal exporters [45, 46]. Indeed, numerous transporters localized to lysosomal and late endosomal membranes exhibit strong pH dependence, as the acidic lumen regulates their structural conformation, substrate affinity, and transport activity [20, 47, 48, 49].

For example, cystinosin (SLC66A1), a lysosomal transporter that exports cystine into the cytosol, relies on a highly pH‐dependent, proton‐coupled mechanism. pH changes induce conformational shifts in cystinosin, suggesting that protonation of specific residues facilitates transport [50]. Notably, not all lysosomal transporters couple their activity directly to the proton gradient. PQLC2, a PQ‐loop repeat‐containing protein that mediates efflux of cationic amino acids, also requires the acidic environment for activity [51], but its transport is uncoupled from the proton gradient [4].

SLC38A9, a low‐activity lysosomal arginine transporter, differs from classical high‐capacity transporters in that its relatively weak transport efficiency enables it to serve as both a transporter and a nutrient sensor (“transceptor”) for mTORC1 activation [14]. This dual role necessitates tight regulation by the intracellular environment. To investigate how such regulation is achieved, we focused on the influence of pH on SLC38A9 activity and sought to identify the molecular determinant responsible for this pH‐dependent modulation.

Here, we demonstrate that SLC38A9 is activated by pH but relies on the sodium gradient to drive transport. Our study identified His544 as the critical residue responsible for pH sensing in SLC38A9. Using site‐directed mutagenesis, we systematically mutated conserved histidine residues. Arginine uptake assays showed that three of the four conserved histidines (His339, His373, and His480) are not required for pH‐dependent activity, as their mutants retained pH‐sensitive transport (Fig. S2). In contrast, His544 is essential: the H544A mutant completely lost pH sensitivity. Similarly, a quadruple histidine mutant (4H) lacking all four conserved histidines showed no pH dependence, whereas reintroduction of His544 (3H mutant) restored pH‐dependent arginine uptake (Fig. S2). Importantly, His544 primarily mediates pH regulation without substantially affecting transport itself, as both the H544A and 4H mutants retained baseline arginine transport activity. These findings indicate that His544 does not directly participate in substrate binding but instead functions as a pH‐sensitive regulatory site. Thus, local ionic changes across the lysosomal membrane likely regulate arginine transport by inducing allosteric changes in SLC38A9.

The previous biochemical characterization by Scalise *et al*., [34] (2019) has established that SLC38A9 transports some amino acids using the transmembrane pH gradient across the lysosomal membrane. Our work builds upon this foundation by providing a structural and mechanistic framework. Our observation of pH‐dependent arginine transport contrasts with the findings of Scalise *et al*., [34], likely due to differences in experimental setup and lipid composition. While Scalise *et al*., [34] utilized a “right‐side‐out” orientation that effectively forced SLC38A9 to act as an importer at pH 5.5, our assay was optimized to mimic its physiological role as a lysosomal exporter. Furthermore, we utilized a neutral Egg‐PC environment rather than a heterogeneous 10% egg yolk phospholipid mixture; we propose that this zwitterionic environment better preserves the conformational flexibility required for the His544‐mediated regulation mechanism.

We demonstrate that His544 acts as an intrinsic pH sensor that allosterically modulates the transport activity of SLC38A9. At the neutral pH (7.2), the imidazole ring of His544 is unprotonated. In this condition, the aromatic ring stabilizes nearby protonated side chains through cation‐π stacking, most prominently with the conserved Arg167 in the human protein (Arg155 in the zebrafish ortholog) [22]. This contact anchors transmembrane helices 2 and 10 in a conformation that favors substrate binding (Fig. 5A).

**Fig. 6:**
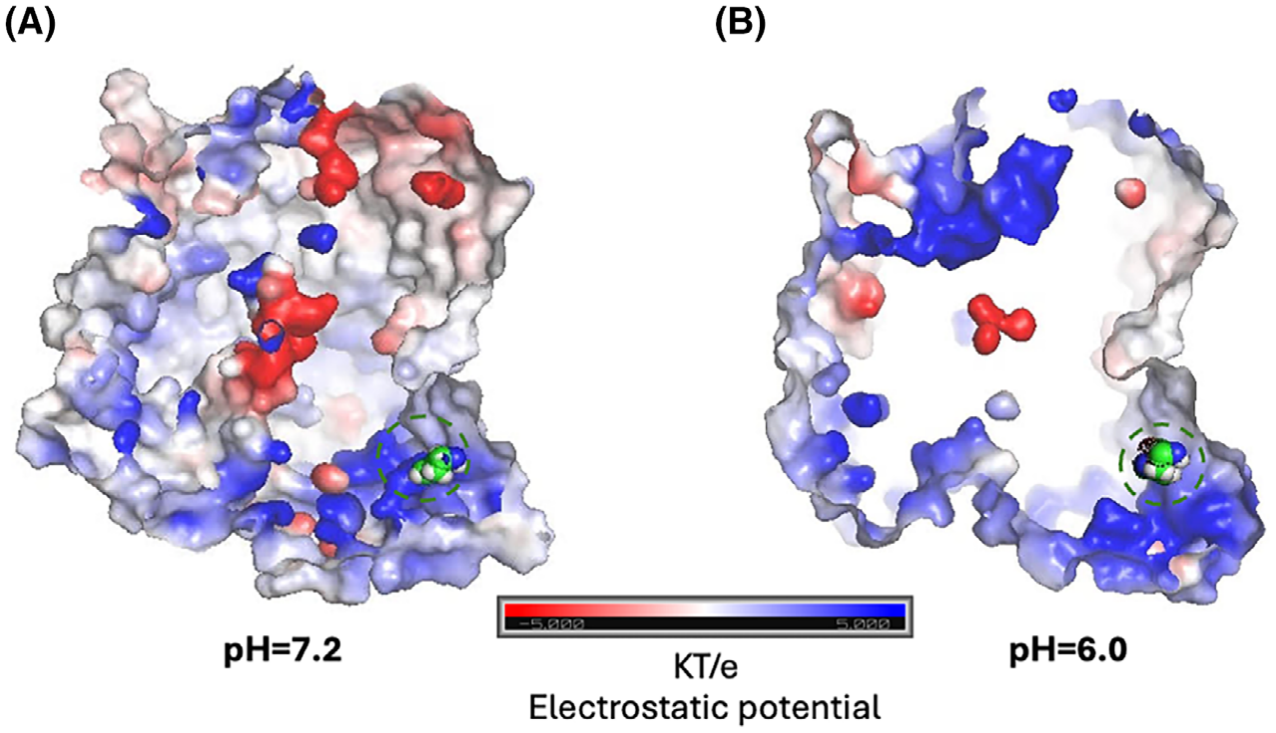
Comparison of drSLC38A9 electrostatic potential at high and low pH, revealing the pH‐sensing role of His532. Electrostatics maps for drSLC38A9 structure in different pH. (A) Electrostatic cross‐sectional view of the drSLC38A9 under high pH; (B) Electrostatic cross‐sectional view of the drSLC38A9 under low pH. Locations of His532 are outlined by a dashed green circle.

When the lumen acidifies to pH 6.0, His544 becomes protonated and gains a positive charge (Fig. 6). The former attractive interaction with Arg167 turns repulsive and the original clamp is released [22]. Protonated His544 can now form new cation‐π or hydrogen bond contacts with neighboring aromatic or polar residues—Tyr163 and Ser523 in the human protein, Tyr151 and Ser511 in the zebrafish protein—which realign transmembrane helices 2 and 10 into the inward open, substrate‐free conformation observed at the low pH (Fig. 5B). A similar mechanism was proposed for other amino acid transporters such as AdiC [52], SNAT2, and SNAT5 [31].

Because these two transmembrane helices form the principal gates that occlude or release arginine during the transport cycle (as seen in AdiC [52]), this pH‐dependent interaction modulation provides a direct molecular link between luminal acidity and transport kinetics. The conservation of His544 and its Arg, Tyr, and Ser partners across SLC38A9 homologs supports an evolutionarily preserved mechanism. Alternating protonation states of His544 therefore rewire its local interaction network and drive the structural transitions that underlie arginine uptake and signaling by SLC38A9. Therefore, we suggest that His544 serves as a pH‐dependent allosteric modulator that fine‐tunes transport efficiency. Rather than acting as a simple “on/off” switch, this residue provides allosteric control by alternating its exposure between the cytosolic and luminal compartments during the transport cycles. The local environment around His544 within the SLC38A9 likely shifts its p*K*_a_. When the bulk cytosolic pH is 7.2, the electrostatic environment near the membrane‐aqueous interface can significantly alter the protonation state of this histidine residue. Our high‐resolution structures at varying pH levels also suggest that this His544 hydrogen‐bond network is sensitive to the pH changes, which involve the structural rearrangements in TM2, TM10, and TM 11. By sensing the pH gradient through these alternating conformational shifts, SLC38A9 couples transport efficiency to lysosomal acidification. This conformation sensitivity ensures that the SLC38A9 remains active only when a sufficient pH difference exists, preventing amino acids “leakage” if lysosomal acidification is compromised.

The orientation of SLC38A9 within our proteoliposomes likely follows a mixed (approximately 50/50) distribution, as is typical for detergent‐mediated reconstitution using Bio‐Bead removal [53]. While the alternative protocol may achieve uniform orientation under specific conditions [6], our system was optimized using n‐ocyt‐β‐*d*‐glucoside (OG) to prioritize membrane integrity and complete detergent removal. Despite this randomized orientation, our transport assay (Fig. 4B) demonstrate that arginine uptake is robust under an inward proton gradient (pH 5.5 external/pH 7.5 internal), mimicking the physiological lysosomal‐to‐cytosolic environment. While our current structures (PDB 6C08, 7KGV) capture cytosol‐open states where His544 (His 532 in drSLC38A9) is exposed to a neutral pH, we propose that this residue does not act as a binary “off” switch. Instead, we propose that His544 functions as a pH‐dependent allosteric modulator. With this allosteric modulation, the protonation state of His544 residue dictates conformational stability of SLC38A9: protonation on the acidic luminal side stabilizes the lumen‐open state to facilitate substrate binding (likely involving the TM10 and the TM2 rearrangements), whereas deprotonation upon exposure to the neutral cytosolic face promotes the cytosol‐open state to facilitate arginine release. While this randomized orientation does not alter the qualitative observation of pH sensitivity and sodium dependence, it suggests that our findings should be interpreted as a structural and mechanistic basis for transport, rather than an absolute quantification of physiological flux. Accordingly, we describe the sodium gradient as a major contributor to the transport, while the proton gradient acts as a critical allosteric signal to ensure efficient amino acid export only under proper lysosomal acidification.

While our functional assays utilized a 100% Egg‐PC environment, to isolate the specific effects of the His544‐mediated pH sensor, it is important to consider the role of other lipid components, such as cholesterol. SLC38A9 has been shown to physically interact with cholesterol, which acts as a key regulator of the Ragulator‐Rag complex in response to cholesterol availability [54], and for glutamine uptake [34]. The physical interaction between SLC38A9 and cholesterol might add another layer of regulation for arginine uptake in varied pH conditions. Future studies incorporating cholesterol or molecular dynamics simulation will be needed to determine how cholesterol–SLC38A9 interactions integrate with the pH‐dependent allosteric regulation described here.

Since pH serves as a proxy for cellular metabolism, fluctuations in intracellular pH often reflect changes in metabolic state [55]. Indeed, altered intracellular pH is strongly associated with aging and numerous human diseases, including neurodegenerative disorders and cancer [56, 57, 58]. Although our assays were performed *in vitro*, the pH‐sensing role of His544 holds significant *in vivo* relevance. In a physiological environment, SLC38A9 must navigate a steep pH gradient between the lysosomal lumen and cytosol. Our pH‐dependent allosteric modulation model suggests that His544 ensures arginine export is coupled to this gradient, providing a refined regulatory and quality control mechanism. As SLC38A9 functions as a transceptor in the mTORC1 nutrient‐sensing machinery, the shifting of the His544 hydrogen bond network may also influence the movement of the “N‐plug” in SLC38A9. This “N‐plug” directly interacts with Rag GTPases to initiate mTORC1 activation [14, 15]. Consequently, changes in lysosomal pH could directly modulate the His544 sensor, thereby coordinating the arginine transport cycle with the regulated exposure of the “N‐plug.” Therefore, by sensing pH, SLC38A9 is uniquely positioned to integrate metabolic signals and adjust nutrient uptake in accordance with the cell's physiological state.

## Author contributions

All authors participated in the design of this project. XM performed protein expression, purification, and proteoliposome reconstitution. XM performed functional experiments and data collection. XM, ASH and TG participated in data analysis and figure preparation. All authors wrote the manuscript.

## Supporting information


**Fig. S1.** [3H]‐arginine uptake activity of drSLC38A9 proteoliposomes in the presence of an inward versus outward H+ gradient versus symmetric pH.
**Fig. S2.** Analysis [3H]‐arginine uptake assays of histidine mutants of SLC38A9.

## Data Availability

All data are contained within the manuscript.
