# Supplementary material for: pH‐mediated activation of the lysosomal arginine sensor SLC38A9
Source: FEBS Lett. 2026 May 3;600(13):1859–71. doi: 10.1002/1873-3468.70352 (PMC13358448; doi:10.1002/1873-3468.70352)
Supplement: Supplementary file 1 — Fig. S1. [3H]‐arginine uptake activity of drSLC38A9 proteoliposomes in the presence of an inward versus outward H+ gradient versus symmetric pH. Fig. S2. Analysis [3H]‐arginine uptake assays of histidine mutants of SLC38A9. [file FEB2-600-1859-s001.docx]

**pH-mediated allosteric activation of the lysosomal arginine sensor SLC38A9**

**Xuelang Mu^1,2^, Ampon Sae Her****^1^, Tamir Gonen^1,2,3^***

**Affiliations**

^1^ Departments of Biological Chemistry and Physiology, University of California, Los Angeles, CA, USA.

^2^ Molecular Biology Institute, University of California, Los Angeles, Los Angeles, CA 90095, USA.

^3^ Howard Hughes Medical Institute, University of California, Los Angeles CA, USA.

* To whom correspondence should be sent T.G. [tgonen@g.ucla.edu](mailto:tgonen@g.ucla.edu)

**
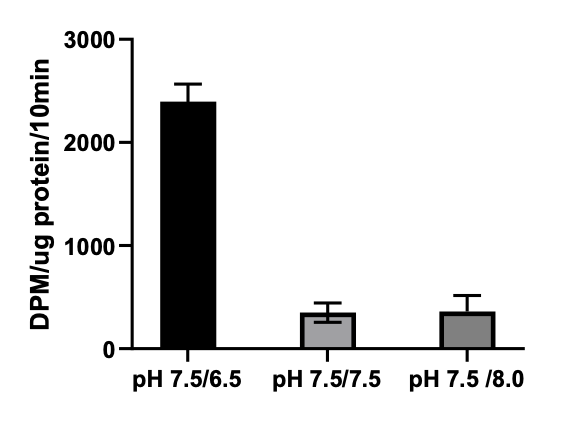
**

**Supplemental Figure S1. [^3^H]-arginine uptake activity of drSLC38A9 proteoliposomes** in the presence of an inward vs outward H^+^ gradient vs symmetric pH. All the experiments had an inward Na^+^ gradient. Error bars, s.e.m. from three independent proteoliposome preparations; *n* = 3 biological replicates. Background uptake by liposome controls (without SLC38A9) were subtracted from these final arginine uptake graphs.


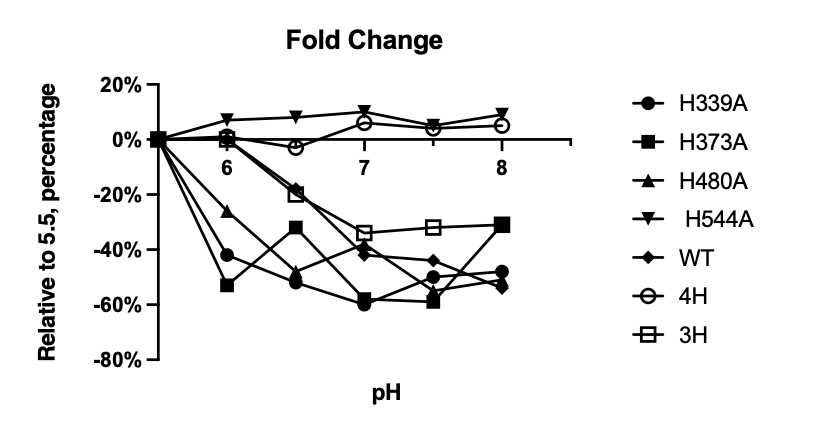


**Supplemental Figure S2. Analysis [^3^H]-arginine uptake assays of histidine mutants of SLC38A9**. Uptake assays of single-site mutants of hSLC38A9 (H339A, H373A, H480A, and H544A) in proteoliposomes are compared to pH 5.5 and plotted as percentage changes in the y-axis. 4H mutant represents the mutant where all 4 histidines were mutated to alanine (H339A, H373A, H480A, H544A). 3H mutant represents the mutant where only 3 histidines were mutated to alanine (H339A, H373A, H480A). The results show the fold change of [^3^H]-arginine uptake at different pH compared to its at pH5.5, in each mutant and the wild-type SLC38A9.
